# Cutting Edge Molecular Therapy for Acute Myeloid Leukemia

**DOI:** 10.3390/ijms21145114

**Published:** 2020-07-20

**Authors:** Kenichi Miyamoto, Yosuke Minami

**Affiliations:** Department of Hematology, National Cancer Center Hospital East, Kashiwa 277-8577, Japan; kenmiyam@east.ncc.go.jp

**Keywords:** acute myeloid leukemia, FMS-like tyrosine kinase 3, isocitrate dehydrogenase, immune checkpoint inhibitor, chimeric antigen receptor

## Abstract

Recently, whole exome sequencing for acute myeloid leukemia (AML) has been performed by a next-generation sequencer in several studies. It has been revealed that a few gene mutations are identified per AML patient. Some of these mutations are actionable mutations that affect the response to an approved targeted treatment that is available for off-label treatment or that is available in clinical trials. The era of precision medicine for AML has arrived, and it is extremely important to detect actionable mutations relevant to treatment decision-making. However, the percentage of actionable mutations found in AML is about 50% at present, and therapeutic development is also needed for AML patients without actionable mutations. In contrast, the newly approved drugs are less toxic than conventional intensive chemotherapy and can be combined with low-intensity treatments. These combination therapies can contribute to the improvement of prognosis, especially in elderly AML patients who account for more than half of all AML patients. Thus, the treatment strategy for leukemia is changing drastically and showing rapid progress. In this review, we present the latest information regarding the recent development of treatment for AML.

## 1. Introduction

Acute myeloid leukemia (AML) is a genetically heterogeneous malignancy of hematopoietic stem cells. Conventionally, the prognosis of AML is determined based on chromosomal abnormalities and fusion genes. Intensive chemotherapy, such as 7 days of cytarabine + 3 days of anthracycline (7 + 3) or high-dose cytarabine, are standard treatments for younger AML patients, and the indication of transplantation is considered based on the prognosis of AML. In contrast, more than half of the patients with newly diagnosed AML are elderly [1] and ineligible for intensive chemotherapy. As a result, the elderly patients (older than 65 years of age) with AML have a short survival (5-year survival estimates (<10%) [2,3], and there is a large unmet need for treatment.

Recently, whole exome sequencing for AML has been performed by a next-generation sequencer in several studies [4,5]. It was revealed that a few gene mutations are identified per AML patient [4,5]. Among them, *FLT3* (28%), *NPM1* (27%), *DNMT3A* (26%), and *IDH1/2* (20%) mutations are observed in 20% to 30% of cases, but the frequency of more than 10 other types of mutations is less than 10% [5]. Some of these low-frequency mutations are actionable mutations, which are defined as genetic aberrations in the DNA and would be expected to elicit a response to an approved targeted treatment that is available for off-label treatment or available in clinical trials [6]. Since 2017, four new drugs targeting gene mutations (midostaurin, giltertinib, ivosidenib, and enasidenib) have been approved by the US Food and Drug Administration (FDA) for AML (Table 1). The era of precision medicine for AML has arrived, and it is extremely important to detect actionable mutations relevant to treatment decision-making.

However, the percentage of actionable mutations found in AML is about 50% at present [5], and therapeutic development is also needed for AML patients without actionable mutations. Indeed, the FDA has approved four drugs (venetoclax, CPX-351, mylotarg, and glasdegib) except for agents targeting actionable mutations. In addition, these newly approved drugs are less toxic than the conventional intensive chemotherapy and can be combined with a low-intensity treatment such as low-dose cytarabine or azacitidine. Therefore, it is expected that these combination therapies will contribute to the improvement of prognosis, especially in elderly AML patients who account for more than half of all AML patients.

**Figure 1:**
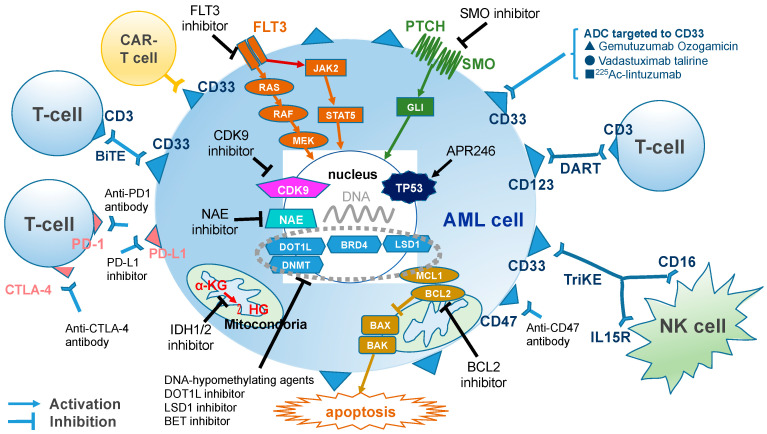
The major targetable pathways and abnormalities in acute myeloid leukemia (AML). Small molecule drugs targeting mutant genes, small molecule drugs targeting signal pathways, drugs targeting epigenetic regulation, antibody therapy, immune checkpoint inhibitors, and adoptive therapy in AML.

Thus, the treatment strategies for leukemia are drastically changing with the rapid development of new drugs. In this review, we provide the latest information regarding the recent developments in AML treatment, including small molecule drugs targeting mutant genes, small molecule drugs targeting signal pathways, drugs targeting epigenetic regulation, antibody therapy, immune checkpoint inhibitors, and adoptive therapy (Figure 1).

Abbreviation; AML, acute myeloid leukemia; ADC, antibody drug conjugate; α-KG, a-ketoglutarate; BAK, BCL-2 antagonist/killer; BAX, BCL-2-associated X protein; BCL2, B-cell leukemia/lymphoma 2; BET, bromodomain and extra-terminal motif; BiTE, bispecific T cell engagers; BRD, bromodomain; CAR, chimeric antigen receptor; CDK, cyclin dependent kinase; CTLA-4, cytotoxic T-lymphocyte-associated protein 4; DART, dual affinity retargeting molecules; DNMT, DNA methyltransferase; DOT1L, disruptor of telomeric silencing-1-like histone methyltransferase; FLT3, FMS-like tyrosine kinase 3; GLI, glioma-associated oncogene; IDH, isocitrate dehydrogenases; IL15R, interleukin 15 receptor; LSD1, lysine-specific histone demethylase 1; MCL1, myeloid cell leukemia 1; NAE, NEDD8-activating enzyme; PD-1, programmed cell death 1; PD-L1, programmed death-ligand 1; PTCH, Patched-1; SMO, smoothened; TriKE, trispecific killer cell engagers; 2HG, R-2-hydroxyglutarate

## 2. Small Molecule Drugs Targeting Mutant Genes

### 2.1. Drugs Targeting fms-Like Tyrosine Kinase 3 Mutation

FMS-like tyrosine kinase 3 (FLT3) belongs to a cytokine receptor (CD135) tyrosine kinase family and regulates the proliferation and differentiation of early hematopoietic stem and progenitor cells [7]. *FLT3* mutations are found in approximately 30% of patients with AML, and two types of mutations in the *FLT3* gene are well-known. *FLT3* internal tandem duplications (ITDs) of the juxtamembrane domain occurs in around 25% of AML patients [8], and point mutations in the activation loop of the tyrosine kinase domain (TKD) occurs in about 5–10% of AML patients [8]. *FLT3*-ITD is a poor prognostic factor based on the *FLT3*-ITD to wild-type (WT) allelic ratio [9,10,11,12,13,14,15,16]. Moreover, a high *FLT3*-ITD to WT allelic ratio (*FLT3*-ITD^high^, ≥0.5) is associated with poor prognosis, but a low *FLT3*-ITD to WT allelic ratio (*FLT3*-ITD^low^, <0.5) is not associated with poor prognosis [17].

First-generation FLT3 tyrosine kinase inhibitors (TKIs), such as midostaurin and sorafenib, were not designed to target FLT3 kinase [18]. However, these inhibitors can show activity against *KIT*, *PDGFR,* and *VEGFR* as multikinase inhibitors [18].

Midostaurin with intensive chemotherapy prolonged the overall survival (OS) (4-year survival rate of 51% vs. 44%, hazard ratio (HR) 0.78; 95% confidence interval (CI), 0.63–0.96; one-sided *p* = 0.009) and event-free survival (EFS) (4-year EFS rate of 28% vs. 21% HR, 0.78; 95% CI, 0.66–0.93; one-sided *p* = 0.002) compared with a placebo with intensive chemotherapy in a randomized placebo-controlled phase III trial in 717 patients (<60 years old) with newly diagnosed *FLT3*-mutated AML (*FLT3*-ITD and *FLT3*-TKD) (RATIFY) [19]. In this study, midostaurin with intensive chemotherapy showed a better efficacy in OS and EFS irrespective of FLT3 mutation status (FLT3-ITD^high^, ≥0.7; FLT3-ITD^low^, <0.7; or FLT3-TKD) [19]. Accordingly, midostaurin was approved in combination with standard chemotherapy by the FDA in 2017.

Sorafenib is another first-generation multi-kinase inhibitor and was approved for several solid tumors. Although the addition of sorafenib to intensive chemotherapy did not show clinical benefits in a phase II trial [20], several studies demonstrated the efficacy of sorafenib as a maintenance therapy post-allogeneic stem cell transplantation for *FLT3*-ITD-positive AML [21,22,23]. In a Sormain trial, a 2-year relapse-free survival of 85% was shown in the sorafenib group vs. a 2-year RFS of 53.3% in the placebo (HR 0.39, 95% CI; 0.18–0.85; *p* = 0.0135) as maintenance therapy post-allogeneic stem cell transplantation for *FLT3*-ITD-positive AML [23]. In settings other than allogeneic stem cell transplantation, sorafenib showed clinical activity in combination with a lower-intensity chemotherapy such as azacitidine (overall response rate (ORR) 46%, complete response (CR) 16%) in patients with *FLT3*-ITD-positive relapsed or refractory AML [24]. 

Second-generation FLT3 TKIs such as quizartinib, crenolanib, and gilteritinib have a more selective inhibition of FLT3 than first-generation FLT3 TKIs. Quizartinib is designed to target FLT3 and a highly selective *FLT3*-ITD inhibitor, but the inhibitory activity against *FLT3*-TKD is low [25,26]. In contrast, quizartinib does not show activity against *FLT3^D835^*-mutated AML [27]. Notable side effects of quizartinib include QT interval prolongation. The frequency of QT interval prolongation was reduced by the administration of lower dose rates (30–60 mg/day) compared with higher doses (90–135 mg/day) while maintaining efficacy [28,29]. In a phase II trial, quizartinib showed single-agent activity (cCR rate of 50% (CR 3% + *C*Ri 47%)) in *FLT3*-ITD-positive relapsed/refractory (R/R) AML patients [30]. There was a high frequency of CR with incomplete hematologic recovery in this study. Poor blood cell recovery may occur due to inhibition against KIT by quizartinib [30]. In a phase III trial (QuANTUM-R study, *n* = 367), quizartinib showed a survival benefit versus (vs.) salvage chemotherapy (median OS of 6.2 months vs. 4.7 months; HR 0.76; 95% CI, 0.58–0.98; *p* = 0.02), with a manageable safety profile in R/R *FLT3*-ITD-positive AML patients [31]. Quizartinib was approved for R/R AML patients with *FLT3*-ITD in Japan but not the USA in 2019.

Crenolanib shows inhibitory activity against both *FLT3*-ITD and *FLT3*-TKD, including *D835* [32]. In a phase II trial of 65 *FLT3*-ITD-positive R/R AML patients treated with crenolanib as a single agent, there was an ORR of 50% (CRi 39%, partial remission (PR) 11%) among 18 patients who had not received prior FLT3 inhibitors and 31% (CRi 17%, PR 14%) among 36 patients who had received prior FLT3 inhibitors [32]. Crenolanib in combination with intensive chemotherapy showed a cCR rate of 83% (24/29) in younger *FLT3*-mutated (ITD and TKD) AML patients (<60 years) [33]. A phase II trial comparing crenolanib vs. midostaurin in combination with induction chemotherapy and consolidation therapy in newly diagnosed AML patients (≤60 years) with the *FLT3* mutation is ongoing (NCT03258931).

Gilteritinib is a highly selective *FLT3* TKI; it also inhibits AXL, which is another receptor tyrosine kinase that promotes proliferation and activates AML cells [34,35]. Gilteritinib showed a cCR rate of 41% and a CR rate of 11% in 169 patients with an *FLT3* ITD or TKD mutation in a phase II trial including 252 R/R AML patients [36]. Gilteritinib as a single agent demonstrated a higher cCR rate (34.0% vs. 15.3%) and longer survival (median OS of 9.3 months vs. 5.6 months; HR for death, 0.64; 95% CI, 0.49–0.83; *p* < 0.001) compared with salvage chemotherapy in the phase III ADMIRAL trial including 247 R/R *FLT3*-mutated AML patients [37]. Based on the results of an interim analysis of this study, gilteritinib was approved by the FDA in 2018. Furthermore, a randomized trial evaluating the additional effect of gilteritinib on midostaurin in combination with intensive chemotherapy in untreated patients (≤65 years) with *FLT3*-mutated AML has been initiated (NCT03836209). Moreover, gilteritinib is currently being studied as an upfront treatment vs. midostaurin in combination with intensive chemotherapy and as a maintenance therapy following induction/consolidation treatment in first remission (NCT02236013 and NCT 02927262). Gilteritinib is also being studied as a maintenance therapy following allogeneic stem cell transplantation for patients with *FLT3*-ITD-positive AML in the phase III setting (NCT02997202).

### 2.2. Drugs Targeting Isocitrate Dehydrogenase Mutation

Isocitrate dehydrogenases (IDH) are enzymes that catalyze the oxidative decarboxylation of isocitrate to a-ketoglutarate (α-KG) [38]. The IDHs are divided into three types. IDH1 is expressed in the cytoplasm and IDH2/3 in the mitochondria. Mutant IDH acquires a new function and produces an oncometabolite called R-2-hydroxyglutarate (2-HG) from α-KG [39]. This conversion reduces the normal α-KG and α-KG-dependent ten-eleven translocation-2 (TET2) function deteriorates. As a result, histone demethylation is not performed correctly, and disorders of cell differentiation occur. 2-HG contributes to cancer by inhibiting various enzymes such as TET and histone demethylase. *IDH1* or *IDH2* mutations occur in 15% to 20% of AML patients, and are more prevalent in AML patients with a normal karyotype [5,40].

Enasidenib inhibits both *R140Q-* and *R172K*-mutated *IDH2* [41]. In a phase I/II trial, 100 mg/d enasidenib showed an ORR of 38.8% with a cCR of 29.0% in 214 patients with R/R *IDH2* mutant AML [42]. The median OS for all 214 R/R AML patients who received enasidenib 100 mg/d was 8.8 months (95% CI, 7.7–9.6). Enasidenib was well tolerated in this study. As a special side effect, IDH differentiation syndrome with fever, dyspnoea due to lung infiltrates, pleural effusion, and leukocytosis occurred in 6.4% of the participating patients [42]. The FDA approved enasidenib for R/R AML with *IDH2* mutations in 2017. Enasidenib combined with intensive chemotherapy achieved a cCR (CRi or CRp) rate of 72% in an open-label, multicenter, phase I study including 89 patients with newly diagnosed AML with an *IDH2* mutation [43]. Currently, a phase III trial evaluating the clinical benefit of enasidenib combined with induction, consolidation, and maintenance therapy for patients with newly diagnosed *IDH2*-mutated AML is ongoing (NCT03839771).

Ivosidenib is a selective *IDH1* mutation inhibitor. Ivosidenib showed an ORR of 41% (CR 22%, CRi 8%) as a single agent in a phase I dose-escalation and dose-expansion study including 258 R/R AML patients with the *IDH1* mutation [44]. The median OS of the primary efficacy population was 8.8 months (95% CI, 6.7–10.2). In this study, IDH differentiation syndrome occurred in 3.9% of the patients who started with an ivosidenib dose of 500 mg daily. Based on the results of this study, ivosidenib was approved by the FDA for newly diagnosed AML with the *IDH1* mutation in patients who are at least 75 years old or who are unfit for intensive chemotherapy on 2 May, 2019. In the frontline setting, ivosidenib (500 mg daily) in combination with intensive chemotherapy showed clinical efficacy (cCR rate of 80%) in a phase I trial of 60 newly diagnosed AML patients with an *IDH1* mutation [43].

Ivosidenib in combinational therapy (intensive or low intensive chemotherapy) is currently being studied in randomized phase III trials investigating previously untreated AML patients with an *IDH1* mutation (NCT03839771 and NCT03173248).

### 2.3. Drugs Targeting TP53 Mutation

The tumor suppressor gene *TP53* has been observed in more than 50% of human cancers, whereas only 5–10% of AML cases have *TP53* mutations [45,46,47]. The frequency of *TP53* mutations is higher in therapy-related AML patients (~30%) and in elderly AML patients with a complex karyotype (~70%) [4,5,48,49]. The *TP53* mutation is an independent indicator of poor outcomes [48]. Intensive chemotherapy with cytotoxic agents, such as anthracyclines and cytarabine, could not overcome the poor outcome (CR rate 20–40%, median OS 4–6 months) of AML with a *TP53* mutation [50,51]. APR-246 is a reactivator of mutated *TP53* [52]. A phase Ib/II study is ongoing to evaluate the safety and efficacy of APR-246 in combination with azacitidine for *TP53*-mutated myeloid neoplasms, including oligoblastic AML (20–30% myeloblasts) (NCT03072043). The preliminary results of this study showed a CR rate of 50% (*n* = 8). Arsenic trioxide/Trisenox/ATO is one of the therapeutic agents that configure the standard treatment of acute promyelocytic leukemia [53]. ATO degrades mutant *TP53* via the 26S proteasome pathway and also activates wild-type *TP53*, leading tumor cells to apoptosis [54,55]. A multi-institution phase II trial is ongoing to identify if using decitabine, cytarabine, and ATO as a therapy for AML patients with *TP53* mutations has a better relapse-free survival and complete response compared to using decitabine and cytarabine (NCT03381781). Furthermore, the cholesterol-lowering drugs named statins (atorvastatin/lipitor) can induce the degradation of abnormal TP53 proteins and inhibit tumor growth in *TP53*-mutated tumor cells [56]. A pilot trial to determine if atorvastatin is sufficient for decreasing the level of conformational mutant *TP*53 in *TP53* mutants and *TP*53 wild-type malignancies, including AML, is ongoing (NCT03560882).

## 3. Small Molecule Drugs Targeting Signal Pathways

### 3.1. BCL2 Inhibition

B-cell leukemia/lymphoma 2 (BCL-2), which is one of the BCL-2 family proteins that also include BCL-XL and MCL-1, promotes cell survival. BCL-2 regulates the mitochondrial apoptotic pathway and plays an important role in the chemoresistance and survival of AML blasts [57,58,59]. Venetoclax is a potent selective inhibitor of BCL-2 but not BCL-XL or MCL-1 [60,61]. In a phase II trial, venetoclax showed an ORR of 19% (6/32, CR 6%, CRi 13%) as a single agent in patients with r/r AML or who are unfit for intensive chemotherapy [62]. An international phase Ib/II study evaluated the safety and efficacy of venetoclax in combination with low-dose cytarabine (LDAC) in elderly patients with previously untreated AML ineligible for intensive chemotherapy [63]. In this study, the CR/CRi rate was 54% (CR 26% + CRi 28%, 95% CI, 42–65%), and the median OS was 10.1 months (95% CI, 5.7–14.2 months) in 82 AML patients who received 600 mg of venetoclax. In contrast, a large, multicenter, phase Ib dose-escalation and expansion study was conducted to evaluate the safety and efficacy of venetoclax in combination with azacitidine or decitabine in elderly patients with previously untreated AML ineligible for intensive chemotherapy (*n* = 145) [64]. The CR/CRi (CR 37%, CRi 30%) rates were 67%, with an ORR of 68% (99/145), and the median OS for all the patients was 17.5 months (95% CI, 12.3 months not reached) in this study. Based on these results, venetoclax was approved in combination with LDAC and hypomethylating agents (HMAs) (azacitidine or decitabine) by the FDA in 2018. In the relapsed and refractory settings, ongoing trials are evaluating the efficacy of venetoclax in combination with FLT3 inhibitors, intensive chemotherapy, or decitabine (NCT03625505, NCT03214562, and NCT03404193). In the front-line setting, several trials assessing the clinical benefit of venetoclax in low intensive or intensive treatments are ongoing (NCT02993523, NCT03069352, NCT03941964, and NCT03709758).

### 3.2. Smoothened (SMO) Inhibitor

The activation of the hedgehog (HH) signaling pathway is known to be involved in leukemia cell survival and drug resistance. Hedgehog, a secreted protein, activates smoothened (SMO) by binding to the PTCH (Patched-1) receptor. Activated SMO initiates GLI (glioma-associated oncogene) protein activation and increased HH target-genes (*BCL2, MYC,* and *Cyclin-D1*) involved in leukemia cell survival and proliferation [65,66]. SMO inhibitors inhibit the HH signaling pathway by binding to SMO. Currently, the FDA approved glasdegib as an oral drug in combination with LDAC for newly diagnosed AML in patients who are 75 years old or older or who have comorbidities that preclude intensive induction chemotherapy. This approval was based on the results of a phase III trial (BRIGHT AML 1003) (*n* = 115) that showed the efficacy of glasdegib + LDAC compared with LDAC alone (median OS of 8.3 months vs. 4.3 months, HR of 0.46 (95% CI: 0.30–0.71; *p* = 0.0002)) in newly diagnosed AML patients unfit for intensive chemotherapy [67]. To evaluate the additional effect of glasdegib on intensive chemotherapy, a phase III study comparing intensive chemotherapy + glasdegib with intensive chemotherapy alone in younger patients with previously untreated AML (BRIGHT AML1019) (*n* = 720) is ongoing (NCT03416179). The other SMO inhibitors (vismodegib and sonidegib) are also being currently investigated in early phase trials (NCT02073838 and NCT01826214, respectively).

### 3.3. Inhibitor of NEDD8-Activating Enzyme (NAE)

The proper expression and degradation of proteins is essential for tumor cell growth and survival. Anti-tumor effects are expected in AML by inhibiting the proteolytic pathway within the proteasome pathway. Pevonedistat (TAK-924/MLN4924), which is a novel inhibitor of NAE, impairs NEDD8-regulated cullin-RING-type ligase action by inhibiting NEDD8, and causes antiproliferative effects [68]. A phase Ib trial of pevonedistat combined with azacitidine for older patients with AML who were deemed unfit to receive intensive chemotherapy has been conducted [69]. In this study, pevonedistat plus azacitidine showed a 50% ORR (20 CR, 5 CRi, and 7 PR), with a median remission duration of 8.3 months (95% CI, 5.52–12.06 months) [69]. A phase III study is currently underway to confirm the utility of pevonedistat plus azacitidine for MDS and AML with a low blast percentage (NCT03268954).

### 3.4. CDK9 Inhibitor

A cyclin-dependent kinase 9 (CDK9) is a member of the CDK family which controls cell-cycle progression and gene transcription. Dysregulation in the CDK9 pathway activates the mRNA transcription of target genes including *MYC* and *MCL-1*, and this has been observed in AML [70]. Alvocidib is a competitive CDK inhibitor of the ATP-binding site with potent activity against the CDK family, including CDK9. Several clinical studies have investigated alvocidib in combination with cytarabine and mitoxantrone (FLAM) in R/R AML [71,72] and newly diagnosed AML [73,74,75]. Overall, CR rates (CR + CRi) of 67% to 70% were achieved in a few phase II trials in newly diagnosed AML patients. To predict patients who respond to alvocidib, a biomarker-driven phase II study comparing FLAM vs. cytarabine and mitoxantrone in patients with *MCL-1*-dependent R/R AML is ongoing (NCT02520011). Outside of that, there is an ongoing phase I study which was initiated to explore alvocidib and standard 7 + 3 chemotherapy in patients with newly diagnosed AML (NCT03298984). The other CDK9 inhibitors (BAY 1143572 and TG02) are also being investigated in early phase trials (NCT02345382, NCT01204164).

## 4. Drugs Targeting Epigenetic Regulation

The system that controls gene expression by chromosomal changes but not changes in the DNA base sequence is called epigenetics. Chromosomal changes refer to chemical modifications, such as the methylation of DNA in nucleosomes, histone acetylation and methylation, and chromatin modification. The Cancer Genome Atlas Research Network reported that, of 200 AML patients, DNA methylation-related genetic mutations occurred in 44% and chromatin modification-related genetic mutations occurred in 30% [5].

### 4.1. DNA-Hypomethylating Agents

Azacitidine or decitabine, which was the first class of epigenetic drug used as DNA-hypomethylating agents, was approved by the FDA in the 2000s. They inhibit DNA methyltransferase, which suppresses tumor suppressor genes by the methylation of the CpG island in the promoter region. These drugs have been used for the treatment of high-grade myelodysplastic syndrome and AML with a low blast count [76,77,78]. In 2018, the FDA approved azacitidine or decitabine in combination with venetoclax for elderly AML patients ineligible for intensive chemotherapy.

In addition to DNA-hypomethylating agents, chromatin modulators have recently been developed as an epigenetic therapy for AML. The three key targets to inhibit are the function of the epigenetic writers (disruptor of telomeric silencing 1-like histone methyltransferase (DOT1L)), epigenetic erasers (lysine-specific histone demethylase 1 (LSD1)), and epigenetic readers (the bromodomain and extra-terminal motif (BET)) in AML patients.

### 4.2. DOT1L Inhibitor

DOT1L is a lysine methyltransferase that methylates a specific amino acid on histone H3K79 and activates a cancer-promoting gene via an MLL fusion protein (MLL-AF9) [79,80]. Pinometostat (EPZ-5676) is a first-in-class inhibitor of the histone methyltransferase DOT1L. Pinometostat showed a modest clinical activity in a phase I study including 43 R/R AML patients [81]. In this study, 37 out of 43 AML patients had *MLL* rearrangements or *MLL* partial tandem duplication, and only one patient out of the 43 AML patients achieved CR.

### 4.3. LSD1 Inhibitor

LSD is a lysine demethylase that catalyzes the demethylation of dimethyl and monomethyl forms of H3K4 and regulates gene expression epigenetically. LSD1, one of the LSD isozymes, is involved in the proliferation of various cancer cells [82,83]. Numerous LSD1 inhibitors, such as TCP, INCB059872, and IMG-7289, are currently being investigated in early clinical trials in AML patients (NCT02273102, NCT02261779, NCT02717884, NCT02712905, and NCT02842827).

### 4.4. BET Inhibitor

BET inhibitors suppress cancer cell growth by inhibiting the binding of bromodomain proteins to histones with acetylation modification. BET inhibitors induce the apoptosis of MLL-fusion leukemia cells [84]. Some clinical trials evaluating the efficacy and safety of BET inhibitors, such as MK-8628-005, FT-1101, and RO6870810/TEN-010, are ongoing (NCT02698189, NCT02543879, and NCT02308761).

## 5. Antibody Therapy

Monoclonal antibodies (MoAb) play an important role in cancer treatment, and it is believed that treatment strategies using MoAb are reasonable for leukemia because of the accessibility of malignant cells in the blood and bone marrow. Surface antigens targeted by MoAb are limited for leukemia, because most of the suitable antigens on AML for MoAb are also found on healthy myeloid precursors, which can easily result in severe cytopenia. Most MoAbs target CD33 or CD123 in clinical studies of AML. MoAb conjugated with a toxic agent (antibody drug conjugates; ADC) has been developed, because unconjugated MoAb is ineffective for AML. Furthermore, MoAbs that bind to both immune cells and leukemia cells have been developed as a novel approach. These MoAbs bring cytotoxic T cells (by binding to CD3) in proximity with leukemia cells (by binding to a specific leukemia antigen) and T cell activation and leukemia cell destruction. This includes bispecific T cell engagers (BiTEs), bispecific/trispecific killer cell engagers designed to target CD16 on NK cells (BiKE/TriKE), or dual-affinity retargeting (DART) molecules.

### 5.1. Antibody Drug Conjugates (ADC)

CD33 is a myeloid differentiation antigen and is expressed in about 90% of leukemic cells [85,86]. Gemtuzumab ozogamicin (GO) is a humanized anti-CD33 antibody conjugated to calicheamicin, which is a cytotoxic agent that causes double-strand DNA breaks. In 2017, the FDA approved GO for the treatment of newly diagnosed CD33-positive AML in adults and for the treatment of R/R CD33-positive AML in adults. This approval is based on the results of ALFA-0701, AML-19, and MyloFrance-1 [87,88,89]. In the ALFA-0701 study, which was a multicenter, randomized, open-label phase III study of 271 patients with newly diagnosed, de novo AML aged 50 to 70 years, the estimated median EFS was 17.3 months in the GO plus chemotherapy group compared with 9.5 months in the chemotherapy-alone group (hazard ratio of 0.56 (95% CI: 0.42–0.76)) [87]. Mylotarg (GO) was reapproved by the FDA for the treatment of adults with newly diagnosed CD33-positive AML and patients aged 2 years and older with R/R CD33-positive AML on 1 September 2017. The FDA extended the indication of mylotarg for newly diagnosed CD33-positive AML to include pediatric patients aged 1 month and older on 16 June 2020.

Another ADC targeting CD33 is vadastuximab talirine (SGN-CD33A) using a pyrrolobenzodiazepine dimer. In a phase I trial (NCT01902329) (*n* = 131), vadastuximab talirine as monotherapy showed clinical activity (cCR 28% (CR 11% + CRi 17%); 95% CI, 9.5–53.5%, 5 of 18 patients) at the recommended monotherapy dose of 40 µg/kg [90]. In a combination cohort (NCT01902329), vadastuximab talirine and HMA had a composite CRR of 70% (CR 43% + CRi 26%; 95% CI, 55.7–81.7%) in all 53 patients [91]. Furthermore, in a phase Ib combination trial (NCT02326584) (*n* = 42), vadastuximab talirine and intensive chemotherapy (7 + 3 chemotherapy) showed a promising efficacy, with a 78% CR and CRi rate [92]. ^225^Ac-lintuzumab (Actimab-A) is a radioimmunoconjugate composed of ^225^Ac linked to a humanized anti-CD33 monoclonal antibody that is currently being studied in untreated older AML patients unfit for intensive chemotherapy. In a phase II study, ^225^Ac-lintuzumab showed a 56% CRR (CRp 22% and CRi 34%) in nine patients [93].

CD123 is expressed in over 95% of AML patient samples, and the overexpression of CD123 is a driver of AML proliferation [94]. CSL360 is a monoclonal antibody targeted to CD123, and this antibody did not show clinical activity in a phase I trial (NCT00401739) [95]. However, several phase I/II clinical trials of anti-CD123 ADCs (NCT02848248) are ongoing based on promising results in preclinical studies [96,97,98].

### 5.2. Antibody-Dependent Cellular Cytotoxicity Therapy 

Recently, novel antibody-based immunotherapies, such as a modified antibody designed to crosslink tumor cells with immune cells (T cells or NK cells), have been developed in AML. BiTE is an antibody drug that combines an antibody against a tumor cell surface antigen and an antibody against an antigen expressed on immune cells, such as CD3. BiTE is already being developed as a treatment for patients with B-cell acute lymphoblastic leukemia (ALL). Blinatumomab, which has dual specificity for CD19 and CD3, showed a high response and relapse-free survival in R/R CD19-positive ALL patients [99,100,101]. In AML, the therapeutic development of some BiTEs is underway. Due to promising preclinical data, several CD33/CD3 BiTE antibodies, such as AMG 330, GEM333, AMG 673 (a half-life extended antibody), and AMV564 (in combination with pembrolizumab), are being investigated in phase I clinical trials in AML patients (NCT02520427, NCT03516760, NCT03224819, and NCT03144245).

DART is a dual-affinity re-targeting molecule that incorporates two single-chain variable fragments (scFv) stabilized by a C-terminal disulfide bridge, while BiTE is a small molecule comprising two scFvs linked in tandem [102,103,104]. DART has additional stability from its disulfide bridge, leading to more favorable cross-linking. A few CD123/CD3 DART antibodies (MGD006 or JNJ-63709178) are being investigated in a phase I clinical trial in R/R AML patients (NCT02152956, NCT02715011).

Similar to BiTE and DART antibodies, bi- and tri-specific killer engagers (BiKEs and TriKEs, respectively) against tumor antigens to activate NK cell cytotoxicity have also been developed. GTB-3550 (161533) is a CD16/IL-15/CD33 TriKE antibody. A phase I/II clinical trial of GTB-3550 is currently underway in CD33-expressing myeloid malignancies, including AML patients (NCT03214666).

## 6. Immune Checkpoint Inhibitor

Immune checkpoint inhibitors have been already approved for several solid tumors by the FDA. In hematologic malignancies, immune checkpoint inhibitors have shown effectiveness in Hodgkin lymphoma and have been approved [105]. Several immune checkpoint pathways, such as cytotoxic T-lymphocyte-associated protein 4 (CTLA-4), programmed cell death protein 1 (PD-1), and macrophage checkpoint CD47, can play an important role in the treatment of AML or MDS.

### 6.1. Anti-CTLA-4

CTLA-4 (CD152) is a protein receptor on T-cells which downregulates immune responses. CTLA-4 acts as an off switch by competing with the costimulatory receptor CD28 for CD80 and CD86 on the surface of antigen-presenting cells, and inhibits T-cell maturation and differentiation [106]. In AML, ipilimumab (anti-CTLA-4 antibody) showed a CR rate of 23% (5/22), with a median 1-year OS rate of 49% in a phase I trial (*n* = 28), including 12 patients with relapsed AML after allogeneic stem cell transplantation [107]. Several clinical trials evaluating the efficacy and safety of ipilimumab are ongoing in AML patients (NCT02890329).

### 6.2. Anti-PD-1

PD-1 is a cell surface molecule that inhibits T-cell proliferation, cytokine production, and cytolytic function by binding to its ligands PD-L1 or PD-L2 on the surface of antigen-presenting cells [108]. In a pilot phase II study (*n* = 14) to evaluate the efficacy of nivolumab maintenance in high-risk AML patients in CR after induction and consolidation chemotherapy, the 6- and 12-month rates of CR duration were 79% and 71%, respectively (NCT02532231) [109]. In a phase I/II study (*n* = 51), a CR/CRi rate of 18% (6/35) with a median OS of 9.3 months (1.8-14.3 months) was shown in R/R AML patients with poor risk features (secondary AML, poor risk cytogenetics) who received nivolumab (anti-PD-1 antibody) and azacitidine approximately every 4–5 weeks indefinitely [110]. Currently, several clinical trials using PD-1 inhibitors in combination with ipilimumab or hypomethylating agents are ongoing in AML patients (NCT02275533, NCT02532231, NCT02464657, NCT02397720, NCT03092674, NCT02768792, NCT02845297, NCT02996474, NCT02708641, NCT02771197, NCT02775903, NCT02892318).

### 6.3. Anti-CD47

CD47 is a cell transmembrane protein that inhibits phagocytosis by interacting with signal regulatory protein-α on antigen-presenting cells [111]. The upregulation of CD47 is found in various types of cancer, including AML, and plays a role in immune escape. In AML, there are some ongoing clinical trials to evaluate the efficacy and safety of Hu5F9G4, which is a monoclonal anti-CD47 antibody for R/R AML patients (NCT02678338, NCT03248479).

## 7. Adoptive Cell Therapy

### Chimeric Antigen Receptor (CAR) T-Cell Therapy

Chimeric antigen receptor (CAR) consists of an extracellular domain generated by joining the heavy and light chain variable regions of a monoclonal antibody with a linker to form an scFv molecule. CAR T-cells are genetically engineered to express CARs on autologous T-cells by a retro-, adeno-, or lentiviral vector carrying the CAR gene, and are infused back into the patient. CAR T-cells combine the antibody in its antigen on the surface of target cells and show tumor-lytic activity.

In the setting of B-ALL and non-Hodgkin’s lymphoma, many clinical trials to evaluate the efficacy of CAR T-cell therapy have been conducted and have shown remarkable clinical activities [112,113,114]. While CAR T-cell therapy shows a high efficacy when the specific antigens on target cells are clearly identified as B-cell malignancies targeting CD19 and CD20 [113,114,115,116], the development of CAR T-cell therapy in AML may be a challenge because AML cells do not have a specific antigen. Many of the AML-associated antigens such as CD33 and CD123 are to some degree expressed on normal myeloid cells, so myeloablation should occur with the use of CAR T-cell therapy targeting CD33 and CD123 [117]. A few clinical trials could not show a high efficacy despite the promising results of preclinical studies of CAR T-cell therapy [118,119]. Besides myeloablation, severe cytokine release syndrome has been reported frequently in CAR T-cell therapy for AML [119,120].

Although there are some challenges, several early phase trials of CAR T-cell therapy targeting AML-associated antigens such as CD33, CD38, CD56, and CD123 are currently ongoing (NCT03971799, NCT04318678, NCT03222674, NCT03190278, NCT03556982, NCT03114670, NCT02159495).

## 8. Conclusions

A lot of clinical trials evaluating the efficacy of promising investigational drugs in AML are ongoing (Table 2), and more drugs will go to market than ever before. Several new agents can create overlapping treatment options, especially in elderly, unfit AML patients as well as in R/R AML patients. From now on, how to use these new agents properly is one of the issues in the treatment of AML. Physicians should select an optimal treatment depending on factors such as age, performance status, comorbidities, and genetic mutations. In particular, genome profiling analysis upon new diagnoses will be needed to select an optimal first line treatment.

**Figure 2:**
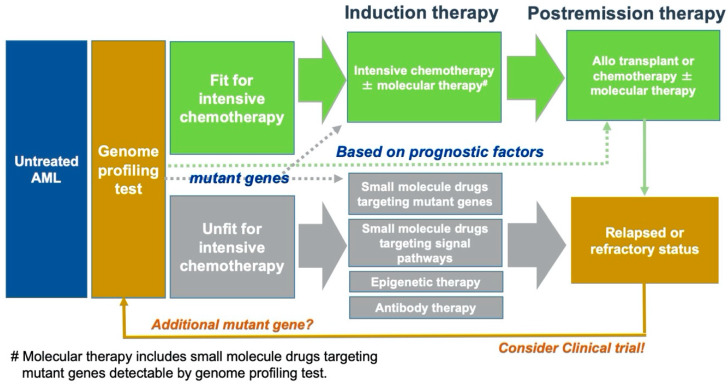
Proposed treatment strategy in AML.

During treatments such as small molecule drugs targeting mutant genes, leukemia cells acquire secondary resistance (e.g., the acquisition of new gene mutations, secondary mutations in the same gene, and new alterations in signaling pathways) [121,122,123,124,125]. Not only upon new diagnoses but also at relapse or refractory periods, genome profiling analyses should be conducted to detect the secondary resistance of leukemia cells and select an optimal second line treatment in AML patients (Figure 2). In the future, more detailed secondary resistance mechanisms and the frequency of secondary resistance will be revealed [126].

Not only upon new diagnoses but also in relapse or refractory periods, genome profiling analyses should be conducted to detect the secondary resistance of leukemia cells and select an optimal second line treatment in AML patients. Molecular therapy includes small molecule drugs targeting mutant genes detectable by a genome profiling test.

## Figures and Tables

**Table 1:** The recent FDA-approved agents.

***Newly Diagnosed AML***			
**Drug/Regimen**	**FDA Approval Indication**	**Approval Date**	**Identifier**
Rydapt/midostaurin + IC	FLT3 mutated AML	28 April 2017	NCT00651261
Mylotarg/GO	Adults or pediatric patients ≥ 1 m with newly diagnosed CD33 positive AML	1 September 2017 (Reapproval) 16 June 2020 (FDA extended the indication to pediatric patients ≥ 1 month)	NCT00927498 NCT00372593
Daurismo/glasdegib + LDAC	> 75 y or unfit for IC	21 November 2018 (accelerated approval)	NCT01546038
Venclexta/venetoclax + HMA	New AML ≥ 75 y or unfit	21 November 2018 (accelerated approval)	NCT02203773
Venclexta/venetoclax + LDAC	New AML ≥ 75 y or unfit	21 November 2018 (accelerated approval)	NCT02287233
Tibsovo/ivosidenib	New AML ≥ 75 y or unfit with IDH mutation	2 May 2019	NCT02074839
***Relapsed/Refractory AML***			
**Drug/Regimen**	**FDA Approval Indication**	**Approval Date**	**Identifier**
Mylotarg/GO	Adults or pediatric patients ≥ 2 y with R/R CD33 positive AML	1 September 2017 (Reapproval)	-
Tibsovo/ivosidenib	R/R IDH1 mutated AML	20 July 2018	NCT02074839
Idhifa/enasidenib mesylate	R/R IDH2 mutated AML	1 August 2017	NCT01915498
Xospata/gilteritinib fumarate	R/R FLT3 mutated AML	28 November 2018	NCT02421939

Abbreviation; IC, intensive chemotherapy; GO, Gemtuzumab ozogamicin; HMA, hypomethylating agents; LADC, low dose cytarabine; R/R, relapsed or refractory.

**Table 2:** Selected ongoing trials for AML featuring the targeted agents.

***Small Molecule Drug Targeting Mutant Genes***							
**Drug**		**Targets**	**Subject**	**Phase/N**	**Investigation**	**Initiation Date/Status**	**Identifier**
Crenolanib		FLT3	Untreated FLT3 + AML	III/510	Crenolanib + IC vs. Midostaurin + IC	Aug 2018/Recruiting	NCT03258931
Gilteritinib		FLT3	Untreated FLT3 + AML	II/179	Gilteritinib + IC vs. Midostaurin + IC	Dec 2019/Recruiting	NCT03836209
Gilteritinib		FLT3	Untreated AML	I/80	Gilteritinib + IC (IDR/AraC or IDR/AraC/DNR)	Jan 2015/Active, not recruiting	NCT02236013
Gilteritinib		FLT3	FLT3 + AML	II/98	Gilteritinib vs. Placebo as maintenance therapy following IC	Jan 2017/Active, not recruiting	NCT02927262
Gilteritinib		FLT3	FLT3 + AML fit for allogeneic SCT	III/346	Gilteritinib vs. Placebo as maintenance therapy following allogeneic SCT	Jun 2017/Recruiting	NCT02997202
Ivosidenib/Enasidenib		IDH1/2	Untreated IDH1/2+ AML/MDS	III/968	Ivosidenib/Enasidenib + IC vs. Placebo + IC	Mar 2019/Recruiting	NCT03839771
Ivosidenib		IDH1	Untreated IDH1 + AML	III/392	Ivosidenib + AZA vs. Placebo + AZA	Jun 2017/Recruiting	NCT03173248
APR-246		TP53	TP53 + AML/MDS/MPN	Ib/II/56	APR-246 + AZA	May 2017/Active, not recruiting	NCT03072043
Arsenic trioxide		TP53	TP53 + AML	II/100	Decitabine/Cytarabine/Arsenic trioxide	Not yet recruiting	NCT03381781
Atorvastatin		TP53	AML/Solid tumors	I/50	Atorvastatin	Jul 2018/Recruiting	NCT03560882
***Small Molecule Drug Targeting Signal Pathway***							
**Drug**		**Targets**	**Subject**	**Phase/N**	**Investigation**	**Initiation Date/Status**	**Identifier**
Venetoclax		BCL2	R/R AML	I/52	Venetoclax + Gilteritinib	Oct 2018/Recruiting	NCT03625505
Venetoclax		BCL2	AML	I/II/116	Venetoclax + Fludarabine/Idarubicin/Cytarabine	Sep 2017/Recruiting	NCT03214562
Venetoclax		BCL2	R/R AML	II/280	Venetoclax + Decitabine	Jan 2018/Recruiting	NCT03404193
Venetoclax		BCL2	Untreated AML unfit for IC	III/443	Venetoclax + AZA vs. Placebo + AZA	Feb 2017/Active, not recruiting	NCT02993523
Venetoclax		BCL2	Untreated AML unfit for IC	III/211	Venetoclax + LDAC vs. Placebo + LDAC	May 2017/Active, not recruiting	NCT03069352
Venetoclax		BCL2	Untreated AML unfit for IC	III/60	Venetoclax +AZA or Decitabine	Aug 2019/Recruiting	NCT03941964
Venetoclax		BCL2	Untreated AML	I/64	Venetoclax + IC	Oct 2018/Recruiting	NCT03709758
Glasdegib		SMO	Untreated AML	III/720	Glasdegib + IC vs. Placebo + IC Glasdegib + AZA vs. Placebo + AZA	Apr 2018/Recruiting	NCT03416179
Vismodegib		SMO	R/R AML or AML unfit for IC	II/40	Vismodegib/Ribavirin/Decitabine vs. Vismodegib/Ribavirin	May 2015/Recruiting	NCT02073838
LDE225		SMO	R/R AML	II/70	LDE225	May 2013/Completed	NCT01826214
Pevonedistat		NAE	Untreated AML/MDS/CMML	III/450	Pevonedistat + AZA vs. AZA	Nov 2017/Active, not recruiting	NCT03268954
Alvocidib		CDK9	R/R MCL-1 dependent AML	II/104	Alvocidib/MIT/AraC vs. MIT/AraC	Mar 2016/Terminated	NCT02520011
Alvocidib		CDK9	Untreated AML	I/32	Alvocidib + IC	Sep 2017/Completed	NCT03298984
BAY1143572 (Atuveciclib)		CDK9	R/R AML	I/42	BAY1143572 (Atuveciclib)	Feb 2015/Completed	NCT02345382
TG02 citrate		CDK9	R/R AML or untreated AML (≥ 65)	I/120	TG02 citrate	Aug 2010/Completed	NCT01204164
***Drugs Targeting Epigenetic Regulation***							
**Drug**		**Targets**	**Subject**	**Phase/N**	**Investigation**	**Initiation Date/Status**	**Identifier**
Tranylcypromine(TCP)		LSD1	R/R AML/MDS	I/17	TCP + ATRA	Mar 2015/Active, not recruiting	NCT02273102
TCP		LSD1	R/R AML or untreated AML unfit for IC	I/II/16	TCP + ATRA	Sep 2014/Recruiting	NCT02261779
TCP		LSD1	AML/MDS unfit for standard therapy	I/II/60	TCP/ATRA/AraC	May 2015/Recruiting	NCT02717884
INCB059872		LSD1	R/R AML or untreated AML	I/II/215	INCB059872 + ATRA in R/R AML INCB059872 + AZA in untreated AML	May 2016/Recruiting	NCT02712905
IMG-7289		LSD1	AML/MDS	I/45	LSD1 ± ATRA	Oct 2016/Completed	NCT02842827
MK-8628 (OTX015)		BET	R/R AML/DLBCL	I/9	MK-8628	May 2016/Active, not recruiting	NCT02698189
FT-1101		BET	R/R AML/MDS or untreated AML unfit for IC	I/94	FT-1101 FT-1101 + AZA	Sep 2015/Completed	NCT02543879
RO6870810 (TEN-010)		BET	R/R AML/MDS	I/26	RO6870810	Nov 2014/Completed	NCT02308761
***Antibody Therapy***							
**Drug**	**Targets**		**Subject**	**Phase/N**	**Investigation**	**Initiation Date/Status**	**Identifier**
SGN-CD33A	CD33		CD33 + AML	I/195	SGN-CD33A +HMA	Jul 2013/Completed	NCT01902329
SGN-CD33A	CD33		AML	I/116	SGN-CD33A +IC followed by SGN-CD33A as maintenance therapy	Dec 2014/Completed	NCT02326584
CSL360	CD123		R/R AML or AML unfit for IC	I/40	CSL360	Dec 2006/Completed	NCT00401739
SGN-CD123A	CD123		R/R CD123 + AML	I/17	SGN-CD123A	Aug 2016/Terminated	NCT02848248
AMG330	CD33/CD3		R/R AML	I/100	AMG330	Aug 2015/Recruiting	NCT02520427
GEM333	CD33/CD3		R/R CD33+ AML	I/33	GEM333	Apr 2018/Recruiting	NCT03516760
AMG673	CD33/CD3		R/R AML	I/50	AMG673	Sep 2017/Recruiting	NCT03224819
AMV564	CD33/CD3		R/R AML or untreated AML unfit for IC	I/148	AMV564 ± Pembrolizumab	Mar 2017/Recruiting	NCT03144245
Flotetuzumab (MGD006)	CD123/CD3		R/R AML	I/II/179	Flotetuzumab(MGD006)	Jun 2014/Recruiting	NCT02152956
JNJ-63709178	CD123/CD3		R/R AML or untreated AML unfit for IC	I/120	JNJ-63709178	Jun 2016/Recruiting	NCT02715011
GTB-3550	CD16/IL-15/CD33		R/R CD33 + AML/MDS	I/II/60	GTB-3550	Jan 2020/Recruiting	NCT03214666
***Immune Checkpoint Inhibitor***							
**Drug**	**Targets**		**Subject**	**Phase/N**	**Investigation**	**Initiation Date/Status**	**Identifier**
Ipilimumab	CTLA-4		RR MDS/AM	I/48	Ipilimumab + decitabine	Apr 2017/Recruiting	NCT2890329
Nivolumab	PD-1		Postremission AML	II/82	Nivolumab	May 2015/Active, not recruiting	NCT02275533
Nivolumab	PD-1		AMLwith high risk of relaps	II/30	Nivolumab	Oct 2015/recruiting	NCT02532231
Nivolumab	PD-1		AML/MDS	II/30	Nivolumab and 7 + 3 induction	July 2015/completed	NCT02464657
Nivolumab	PD-1		RR AML, AML > 65 years	II/182	Nivolumab + azacytidine+/-ipilimumab	Apr 2015/recruiting	NCT02397720
Nivolumab	PD-1		Elderly patients MDS or newly diagnosed AML	II/III/1670	Azacitidine+/-nivolumab or midostaurin, or decitabine + cytarabine	Dec 2017/suspended	NCT03092674
Pembrolizumab	PD-1		RR AML	II/37	Pembrolizumab following HDAC salvage induction	Aug 2016/Active, not recruiting	NCT02768792
Pembrolizumab	PD-1		RR MDS/AML and newly diagnosed AML patients (≥ 65)	II/40	Pembrolizumab + Azacitidine	July 2016/recruiting	NCT02845297
Pembrolizumab	PD-1		RR AML	I/II/10	Pembrolizumab + decitabine	Dec 2016/completed	NCT02996474
Pembrolizumab	PD-1		AML patients (≥ 60) in post remission treatment	II/12	Pembrolizumab	Oct 2016/Active, not recruiting	NCT02708641
Pembrolizumab	PD-1		AML patients with high risk of relapse	II/20	Pembrolizumab + Fludarabine/melphalan conditioning + autologous SCT	Sep 2016/recruiting	NCT02771197
Durvalumab	PD-1		High risk MDS, elderly AML patients	II/213	Durvalumab + azacitidine	Jun 2016/Active, not recruiting	NCT02775903
Atezolimumab	PD-1		RR AML, elderly AML patient unfit for IC	I/40	Atezolizumab + guadecitabine	Oct 2016/completed	NCT02892318
Hu5F9-G4	CD47		RR AML, MDS intermediate2 or high risk	I/20	Hu5F9-G4	Nov 2015/completed	NCT02678338
Hu5F9-G4	CD47		RR MDS/AMLor AML/MDS patient unfit for IC	I/257	Hu5F9-G4 + Azacitidine	Sep 2017/recruiting	NCT03248479
***Adoptive Cell Therapy***							
**Drug**	**Targets**		**Subject**	**Phase/N**	**Investigation**	**Initiation Date/Status**	**Identifier**
CAR-T cells	Various (CD33, CD58, CD56, CD123, Muc1)		RR AML	I/II/10	Infusion of Muc1/CD33/CD38/CD56/CD123-specific gene-engineered Tcells	July 2017/recruiting	NCT03222674
CAR-T cells	CD33		RR CD33 + AML	I/II34	FC followed by anti-CD33 CART infusion	Jan 2020/recruiting	NCT03971799
CAR-T cells	CD123		RR CD123 + AML	I/32	FC followed by autologous anti-CD123 CAR-T cells	May 2020/recruiting	NCT04318678
CAR-T cells	CD123		CD123 + AML	I/59	Allogenic anti-CD123 CAR T-cells following lympho depleting regimen	Jun 2017/recruiting	NCT03190278
CAR-T cells	CD123		CD123 + RR AML (> 14)	I/II/10	FC followed by infusion of allogenic or autologous anti-CD123 CAR-T cells	Mar 2018/unknown	NCT03556982
CAR-T cells	CD123		CD123 + AML relapsed after allogeneic SCT	I/20	CD123 CAR-41BB-CD3zeta-EGFRt-expressing Tcells after preconditioning	Mar 2017/recruiting	NCT03114670
CAR-T cells	CD123		RR CD123 + AML or BPDCN (> 12)	I/42	Lymphodepletion with FC, autologous or allogenic CD123 CAR-CD28 CD3 zeta-EGFRt-expressing T lymphocytes	Dec 2015/recruiting	NCT02159495

Abbreviation; IC, intensive chemotherapy; DNR, daunorubicin; AraC, cytarabine; SCT, stem cell transplantation;LADC, low dose cytarabine; HDAC, high dose cytarabine; R/R, relapsed or refractory; ATRA, All-trans retinoic acid; MIT, mitoxantrone; AZA, azacitidine; HMA, hypomethylating agents; MDS, myelodysplastic syndromes; DLBCL, diffuse large B-cell lymphoma; fludarabine + cyclophosphamide.
